# Blunt cerebrovascular injury: incidence and long-term follow-up

**DOI:** 10.1007/s00068-019-01171-9

**Published:** 2019-06-13

**Authors:** Dennis Hundersmarck, Willem-Bart M. Slooff, Jelle F. Homans, Quirine M. J. van der Vliet, Nizar Moayeri, Falco Hietbrink, Gert J. de Borst, Fetullah Cumhur Öner, Sander P. J. Muijs, Luke P. H. Leenen

**Affiliations:** 1 Department of Surgery, University Medical Center Utrecht, Utrecht, The Netherlands; 2 Department of Neurosurgery, University Medical Center Utrecht, Heidelberglaan 100, Post-office 85500, 3508 GA Utrecht, The Netherlands; 3 Department of Vascular Surgery, University Medical Center Utrecht, Utrecht, The Netherlands; 4 Department of Orthopedic Surgery, University Medical Center Utrecht, Utrecht, The Netherlands

**Keywords:** Blunt cerebrovascular injury, Carotid artery injury, Vertebral artery injury, Functional outcomes, Patient-reported outcomes

## Abstract

**Purpose:**

Blunt cerebrovascular injuries (BCVI), which can result in ischemic stroke, are identified in 1–2% of all blunt trauma patients. Computed tomography angiography (CTA) scanning has improved and is the diagnostic modality of choice in BCVI suspected patients. Data about long-term functional outcomes and the incidence of ischemic stroke after BCVI are limited. The aim of this study was to determine BCVI incidence in relation to imaging modality improvements and to determine long-term functional outcomes.

**Methods:**

All consecutive trauma patients from 2007 to 2016 with BCVI were identified from the level 1 trauma center prospective trauma database. Three periods were identified where CTA diagnostic modalities for trauma patients were improved. Long-term functional outcomes using the EuroQol six-dimensional (EQ-6D™) were determined.

**Results:**

Seventy-one BCVI patients were identified among the 12.122 (0.59%) blunt trauma patients. In the first period BCVI incidence among the overall study cohort, polytrauma, basilar skull fracture and cervical trauma subgroups was found to be 0.3%, 0.9%, 1.2%, 4.6%, respectively, which more than doubled towards the third period (0.8, 2.4, 1.9 and 8.5% respectively). Ischemic stroke as a result of BCVI was found in 20 patients (28%). In-hospital stroke rate was lower in patients receiving antiplatelet therapy (*p* < 0.01). Six in-hospital deaths were BCVI related. Long-term follow-up (follow-up rate of 83%) demonstrated lower functional outcomes compared to Dutch reference populations (*p* < 0.01). Ischemic stroke was identified as a major cause of functional impairment at long-term follow-up.

**Conclusions:**

Improved CTA diagnostic modalities have increased BCVI incidence. Furthermore, BCVI patients reported significant functional impairment at long-term follow-up. Antiplatelet therapy showed a significant effect on in-hospital stroke rate reduction.

## Introduction

Blunt cerebrovascular injuries (BCVIs) collectively describe all non-penetrating traumatic injuries to the extra- or intracranial carotid and vertebral arteries. Most of these injuries are found in the extracranial arteries. BCVI is considered a rare cause of cerebral ischemia and ischemic stroke with risk of death or permanent neurological deficits in trauma populations. Short-term observational studies suggest permanent neurological deficits in approximately 50% of BCVI patients, but data about long-term neurological impairment and functional outcomes is still lacking [1, 2].

The most common trauma mechanisms known to cause BCVI are either hyperextension/flexion and rotation of the neck or a direct blow to the blood vessels. These frequently high-energy mechanisms may cause numerous types of injuries, such as cervical spine, maxillofacial and brain lesions, which in some cases are associated with injury to the walls of arteries, called dissection, exposing the underlying collagen and initiating thrombus formation or a hemodynamically significant obstruction. The mortality rates for trauma patients with BCVI are found to be around 20–30% [3–5].

Up to 80% of patients with BCVI do not display neurological deficits at presentation, making the identification of vascular injury difficult [6]. Due to the improved imaging technology and the introduction of standard screening protocols, such as the Memphis and (modified) Denver criteria, the reported incidence of BCVI among blunt trauma patients has increased over recent years [7–11]. The estimated incidence of BCVI among all blunt trauma patients, presenting in high-volume, well equipped trauma centers is around 1–2% [12, 13]. Although aggressive screening has led to the identification of more BCVI cases, debates remain due to costs and potential complications caused by (invasive) screening methods such as angiography [14–16]. Therefore, a standard screening method with high accuracy and low complication rate is needed for an adequate evaluation of the true incidence of BCVI.

Our primary objective was to determine the incidence of BCVI among various subgroups in the blunt trauma population (polytrauma, basilar skull fracture and cervical trauma subgroups), during 10 consecutive years at a level 1 trauma center. Significant changes in imaging modalities or protocols over time, which might affect the found incidence rate, are taken into consideration and recalculated during different time periods. Subsequently, long-term functional outcomes of the BCVI patients were determined.

## Methods

### Setting

This retrospective cohort study was conducted at University Medical Center Utrecht, a level 1 trauma center located in the Netherlands that provides 24-h emergency care for trauma, spine, vascular, and neurosurgery and has interventional radiology services available.

### Patient selection

All trauma patients with BCVI, admitted from the emergency department, from January 2007 to December 2016 were included in this study. All trauma cases that are admitted to hospital are prospectively added to the institutional trauma database by dedicated data managers at our institution. BCVI cases were retrospectively identified by searching in the institutional trauma database according to the corresponding International Classification of Diseases (ICD) 10 and Abbreviated Injury Scale (AIS) codes. Furthermore, records of patients with a high risk of BCVI according to Denver and (modified) Memphis criteria were also separately identified and searched for BCVIs, if not included in the trauma database [7–11]. To minimize the risk of missing cases, an additional search was performed using the Picture Archiving and Communicating System (PACS) to identify unreported BCVI cases based on their high-risk associated lesions (e.g. basilar skull fractures, cervical spine fractures), which were analyzed if not included in the trauma database. Patients younger than 18 years old at time of injury and patients with incomplete medical records were excluded. The total number of patients in blunt trauma population subgroups (polytrauma, basilar skull fracture, and cervical trauma), which had presented at the emergency department and were admitted to hospital in the study period, was also derived from the institutional trauma database to calculate incidence. These subgroups were identified using the corresponding ICD and Abbreviated Injury Scale (AIS) codes. The Biffl et al. grading scale, shown in Table 1, was used to grade the severity of BCVIs [7].

**Table 1:** Biffl et al. injury grading scale, used to grade all identified BCVIs [7]

Grade	Description
1	Irregularity of the vessel wall or dissection/intramural hematoma with < 25% luminal stenosis
2	Intraluminal thrombus, raised intimal flap; dissection/intramural hematoma with > 25% luminal narrowing
3	Pseudoaneurysm
4	Complete occlusion of the vessel
5	Transection of the vessel

### Patient Characteristics

All baseline and additional patient characteristics such as injury severity score (ISS), Glasgow Coma Scale (GCS), mechanism of injury, injury grading and treatment, were extracted from medical reports (Table 2). Polytrauma was defined as an ISS ≥ 16.

**Table 2:** Characteristics of all BCVI patients, questionnaire responders and non-responders

	All BCVI patients (*n* = 71)	Responders (*n* = 38)	Non-responders (*n* = 8)
Age (years)			
At trauma	50 [29–68]	50 [29–65]	47 [31–61]
At follow-up	N/A	54 [35–69]	N/A
Male	46 (65)	24 (63)	5 (63)
ISS	26 [16–36]	21 [13–33]	28 [13–38]
Polytrauma	54 (76)	27 (71)	6 (75)
GCS score at admission	14 [3–15]	15 [11–14]	15 [3–15]
Neurological deficits at admission	61 (86)	32 (84)	7 (88)
Brain injury	30 (42)	10 (26)	3 (38)
Intracranial hemorrhage	28 (39)	9 (24)	3 (38)
Thoracic injury	40 (56)	17 (45)	4 (50)
Abdominal injury	13 (18)	6 (16)	2 (25)
Basilar skull fracture	22 (31)	9 (24)	2 (25)
Spine injury^a^			
Cervical	61 (86)	33 (87)	7 (88)
Thoracic	13 (18)	6 (16)	2 (25)
Lumbar	6 (8)	1 (3)	0 (0)
Spinal cord injury	19 (27)	10 (26)	2 (25)
Type of BCVI^a^			
Carotid artery injury	33 (46)	11 (29)	5 (63)
Vertebral artery injury	48 (68)	30 (79)	3 (38)*
Treatment			
Antiplatelet	40 (56)	25 (66)	7 (88)
None	28 (39)	12 (32)	1 (12)
Endovascular	2 (3)	1 (3)	0 (0)
Surgical	1 (1)	0 (0)	0 (0)
Hospital stay (days)	11 [4–29]	12 [7–29]	28 [8–39]
ICU stay (days)	1 [0–7]	0 [0–7]	3 [0–11]
Outcomes			
Total ischemic stroke	20 (28)	8 (21)	3 (38)
Carotid artery stroke^b,c^	11 (15)	2 (5)	3 (38)
Vertebral artery stroke^b,c^	9 (13)	6 (16)	0 (0)
In-hospital ischemic stroke	18 (25)	8 (21)	2 (25)
In-hospital mortality	16 (23)	N/A	N/A
BCVI related in-hospital mortality	6 (8)	N/A	N/A

Data are presented as the number (%) or the median [IQR: 25th–75th percentile]

*ISS* Injury Severity Score, *GCS* Glasgow Coma Scale, *ICU* Intensive care unit

*p < 0.05

^a^Total number and percentage is greater than 100%, since multiple patients suffered from multiple injuries

^b^One patient suffered from ischemia in both the medial and posterior circulation

^c^One patient died due to diffuse cerebral ischemia, suffering from an affected carotid artery, occluded basilar artery and severe intracranial bleeding. It was, therefore, impossible to determine the responsible artery

Thoracic injuries were defined as all thoracic soft-tissue injuries, with or without fractures of the sternum, ribs, scapula and clavicles. Abdominal injuries were defined as all soft-tissue injuries to the abdomen, excluding pelvic injuries. Cervical, thoracic and lumbar spine injuries were defined as all spine fractures, dislocations and discoligamentous injuries with or without associated spinal cord injury. In some cases cervical trauma patients were also identified as polytrauma patients, and were, therefore, included in both groups. Brain injury was defined as all traumatic intracranial injuries, such as intracranial hemorrhage, contusions and diffuse axonal injuries (visible on CTA-scan or (delayed) MRI-scan). Spinal cord injury was defined as damage to the spinal cord at cervical level, causing complete or incomplete neurological deficit. Cerebral neurological deficits at presentation were defined as either caused by cerebral ischemia or by associated brain injury. Ischemic stroke caused by the vascular injury (instead of progression of traumatic brain injury) was defined as a combination of clinical suspicion for stroke, the side and type of the identified BCVI and hypodensities in areas of the brain visible on the initial CTA-scan, supplied by either the middle cerebral artery (carotid artery) or the posterior circulation (vertebral artery). Most ischemic stroke cases (if patients survived the initial few days) were confirmed by a delayed perfusion scan of the brain. Patients suffering from diffuse cerebral ischemia with bilaterally affected arteries and, therefore, poor cerebral circulation were considered ischemic stroke patients. Antiplatelet therapy was defined as treatment initiated before ischemic stroke occurred.

### Diagnostics

At our institution, trauma patients suspected for underlying injuries after sustaining major trauma received full-body CTA-scanning in the studied period. These patients were in most cases clinically suspicious for injuries in at least 3 body regions. This also included trauma patients that were unconscious at presentation (Intoxication, severe (head) injuries, shock, intubation/sedation). Trauma patients that presented between 2007 and 2010 were scanned using a full body CT-angiogram by a Philips Brilliance© 64-slices CT-scanner (Philips Medical Systems, Best, the Netherlands). During the course of 2010 (until 2014), trauma patients received a full body CT-angiogram using a Philips iCT© 256-slices CT-scanner (Philips Medical Systems, Best, the Netherlands). Patients received split bolus intravenous contrast administration during CT-scanning. The initial contrast administration (Ultravist^®^-300, Bayer, Berlin, Germany) served in imaging of parenchymal organs (total body). The second dose, injected 50 s after the initial dose, facilitated imaging of large arteries to visualize any perforation of the vascular wall (blush) or other vascular injury. In 2014 (until 2016), intravenous contrast administration protocol for trauma patients was changed to improve imaging of vascular structures. This included increase of contrast flow during the second administration from 3 to 6 ml/s, providing a higher contrast density. In the period 2014–2016, the same Philips iCT© 256-slices CT-scanner was used. Furthermore, from the year 2014 all patients with isolated traumatic maxillofacial, cervical or head injuries (with no indication for full-body scanning), visible at CT-scanning, received additional CTA-scanning of the cervical area. Before 2014, cervical CTA-scanning after isolated trauma was rarely performed, only when patients were clinically highly suspicions for vascular injuries (based on signs and symptoms). In all cases, both full-body CTA-scanning as well as CTA-scanning of the cervical spine in the case of isolated injury, the circle of Willis on the distal side and the origin of the carotid and subclavian artery from the aorta on the proximal side, were visualized.

The initial diagnosis of BCVI was made by the attending radiology consultant. Findings concerning the cervical arteries were noted in the patients’ records. All additional cases obtained through a manual search using PACS were reanalyzed. Any doubt about the presence or severity of the vascular wall injury was resolved by an expert group, which included an independent neuroradiologist and two spinal surgeons. If no consensus was achieved, the case was designated as no injury and, therefore, discarded for further analysis.

### Incidence

Overall incidence of BCVI among all blunt trauma cases and three trauma population subgroups (polytrauma, basilar skull fracture, and cervical trauma) were calculated. In addition, due to the changes in imaging and contrast administration protocol during the analyzed periods, we recalculated the incidence during the study periods 2007–2009 (I), 2010–2013 (II) and 2014–2016 (III).

### Outcomes and follow-up

In-hospital mortality of all BCVI patients was determined and stratified for cerebrovascular ischemic stroke confirmed by CT. All discharged patients were seen at the outpatient department after 6 weeks and any related cerebrovascular event was noted. Subsequently, the National Personal Records Database was queried to determine post-discharge mortality at 6 weeks and later on during the follow-up in December 2016.

At the end of the follow-up period in 2018 we determined functional outcomes. We interviewed all remaining BCVI patients or, if the patient was unable to communicate via telephone, the primary caregiver. Again, all cerebrovascular related events including details about diagnosis and timing were noted. Functional outcomes were determined by conducting a structured telephone interview, using the EuroQol six-dimensional (EQ-6D™, Rotterdam, the Netherlands) questionnaire. The EQ-6D™ covers six dimensions [mobility, self-care, usual activities, pain/discomfort, anxiety/depression and concentration/memory (cognition)] that are all divided into three levels (no problems, moderate problems, or severe problems) that can be dichotomized into ‘No problems’ and ‘Problems’. Using only five dimensions of the EQ-6D™ questionnaire (all but concentration/memory (cognition)), the EQ-5D™ index scores were calculated using the Dutch tariff [17]. In addition, the EuroQol Visual Analog Scale (EQ-VAS™) was assessed. This score represents a patient’s self-rated health status on a scale from 0 to 100, a score of 0 being the worst imaginable health state and a score of 100 being the best imaginable health state. The Dutch EQ-5D™ population index norm for ages between 45 and 54, (Median and mean age of BCVI patients at follow-up: 54 and 53 years, respectively) which was reported by Janssen et al. was used in our study [18]. Data for the general trauma population control group at our institution were derived from Gunning et al. who included 1870 trauma patients aged ≥ 18 years old who presented at the same institution from January 1st 2007 till December 2012 with a mean age of 54 years [19]. Baseline characteristics of questionnaire responders were compared to non-responders to identify possible differences in trauma burden, potentially affecting the reported functional outcomes and quality of life.

### Statistical analysis

Statistical and descriptive analyses of patient baseline characteristics demographics, outcomes and questionnaire results were performed using IBM SPSS Statistics version 22 (IBM Corp., Armonk, NY). Non-parametric continuous variables are presented as medians with interquartile range (IQR) P25-P75. Categorical data are displayed as numbers with percentages. The EQ-5D™ index scores were compared with the Dutch reference population index norm of the same age, and compared to the general trauma reference population, both using two sample *t* test. Categorical variables were analyzed using Chi-square test. The relation between continuous outcome measures and dichotomous explanatory variables were assessed using the Mann–Whitney *U* test. A *p* value of < 0.05 was considered significant.

## Results

### Baseline characteristics and incidence

**Fig. 1:**
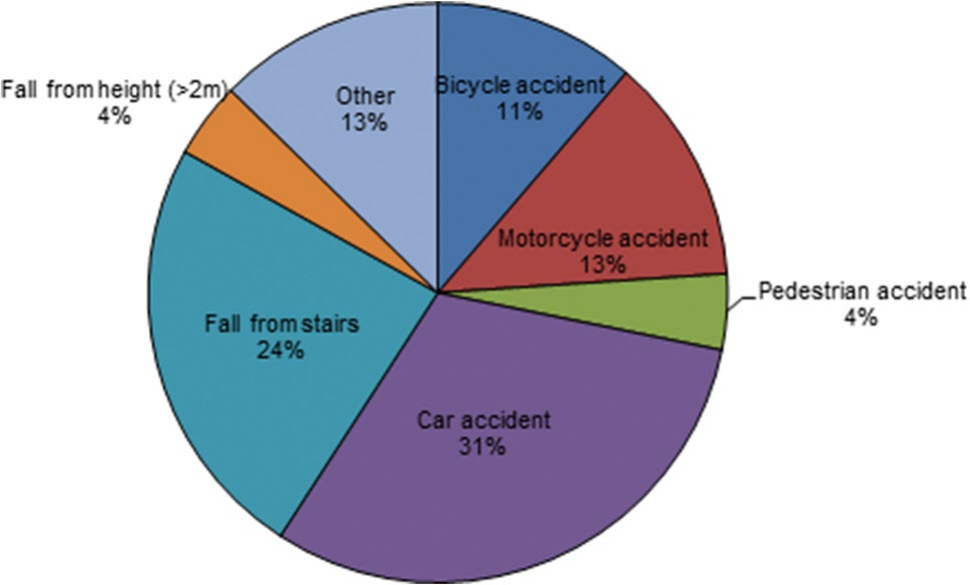
Pie chart displaying the mechanism of trauma of all BCVI patients

Approximately 12.122 blunt trauma patients were admitted to our hospital in the 10-year study period. Seventy-one patients with a total number of 101 BCVIs (0.59%) were identified. Baseline characteristics as well as general and neurological injury severity index are shown in Table 2. Accompanied traumatic brain injury was seen in 42% of all BCVI patients. The most common mechanism of trauma in all BCVI patients was car accidents (31%) followed by fall from stairs (24%) (Fig. 1). Forty-four percent of the BCVIs involved carotid artery injuries, most of which categorized as Biffl et al. grade II as depicted in Table 3 [7].

**Table 3:** Vessel grading according to the Biffl et al. grading scale [7]

Vessel	Grade I	Grade II	Grade III	Grade IV	Grade V	Total (%)
LCA	3	12	6	4	1	26 (26)
RCA	4	6	5	3	0	18 (18)
LVA	2	12	1	17	0	32 (32)
RVA	2	11	0	11	1	25 (25)
Total (%)	11 (11)	41 (41)	12 (12)	35 (35)	2 (2)	101 (100)

Data are presented as the number (%)

*LCA* left carotid artery, *RCA* right carotid artery, *LVA* left vertebral artery, *RVA* right vertebral artery

**Fig. 2:**
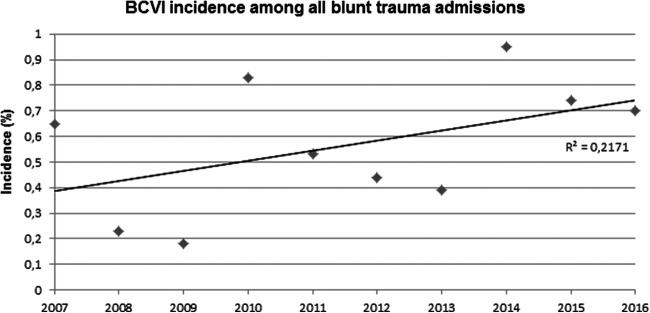
BCVI incidence among all blunt trauma admissions over the 10-year study period

**Fig. 3:**
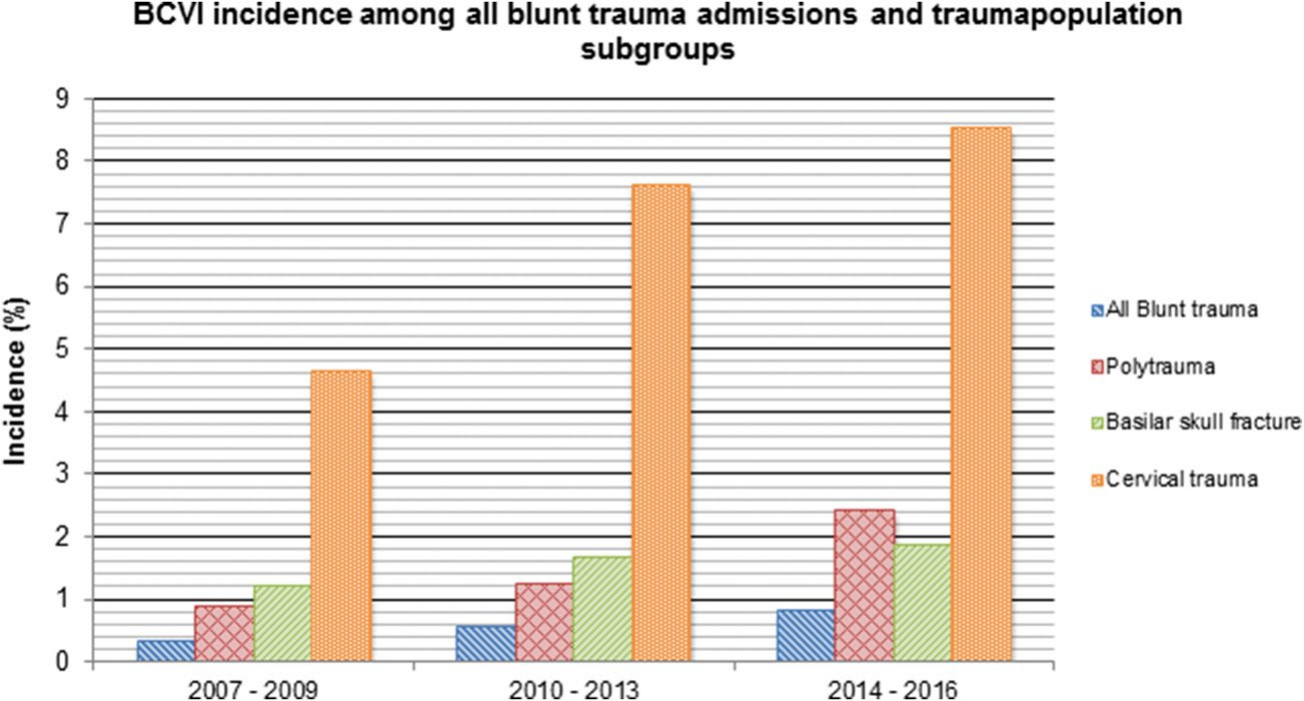
Incidence of BCVI among the overall blunt trauma group, and the 3 trauma population subgroups

In the studied 10-year period, 3529 polytrauma patients, 1359 basilar skull fracture patients and 840 cervical trauma patients were identified among the 12.122 admitted blunt trauma patients. The overall incidence of BCVI among the whole blunt trauma group and polytrauma, basilar skull fracture and cervical trauma subgroups patients during the total study period was 0.59, 1.5, 1.6 and 7.3%, respectively. A gradual increase in BCVI incidence among all blunt trauma admissions as well as the three subgroups was observed (Figs. 2, 3). The cervical spine injury subgroup showed the strongest association with BCVI [Odds ratio (OR) = 88.7, 95% CI 45.3, 173.9], followed by polytrauma (OR = 7.6, 95% CI 4.5, 13.4) and basilar skull fractures (OR = 3.6, 95% CI 2.2, 6.0).

### Outcome and follow-up

Demographics of all BCVI patients, questionnaire responders and non-responders are also shown in Table 2. Sixteen patients died during the initial hospitalization (23%), of which 6 died as a result of BCVI (8%). Eight additional patients died during the follow-up period, after discharge from hospital (11%). Forty of the 71 cases with BCVI received antiplatelet therapy. The decision to start with this therapy depended on the severity of associated injuries, such as ongoing blood loss and severe neurotrauma. During the initial hospitalization, 25% (*n* = 18) of the patients with BCVI suffered ischemic stroke of which 10 were caused by carotid artery injury and 8 by vertebral artery injury. Of these, 3 occurred in the first period (I), 8 occurred in the second period (II) and 7 occurred in the third period (III). Fourteen patients who did not receive antiplatelet therapy due to concomitant injuries, suffered from in-hospital ischemic stroke. The difference in in-hospital stroke rate between patients receiving antiplatelet therapy and those not receiving antiplatelet therapy was found to be statistically significant (*p* < 0.01).

**Fig. 4:**
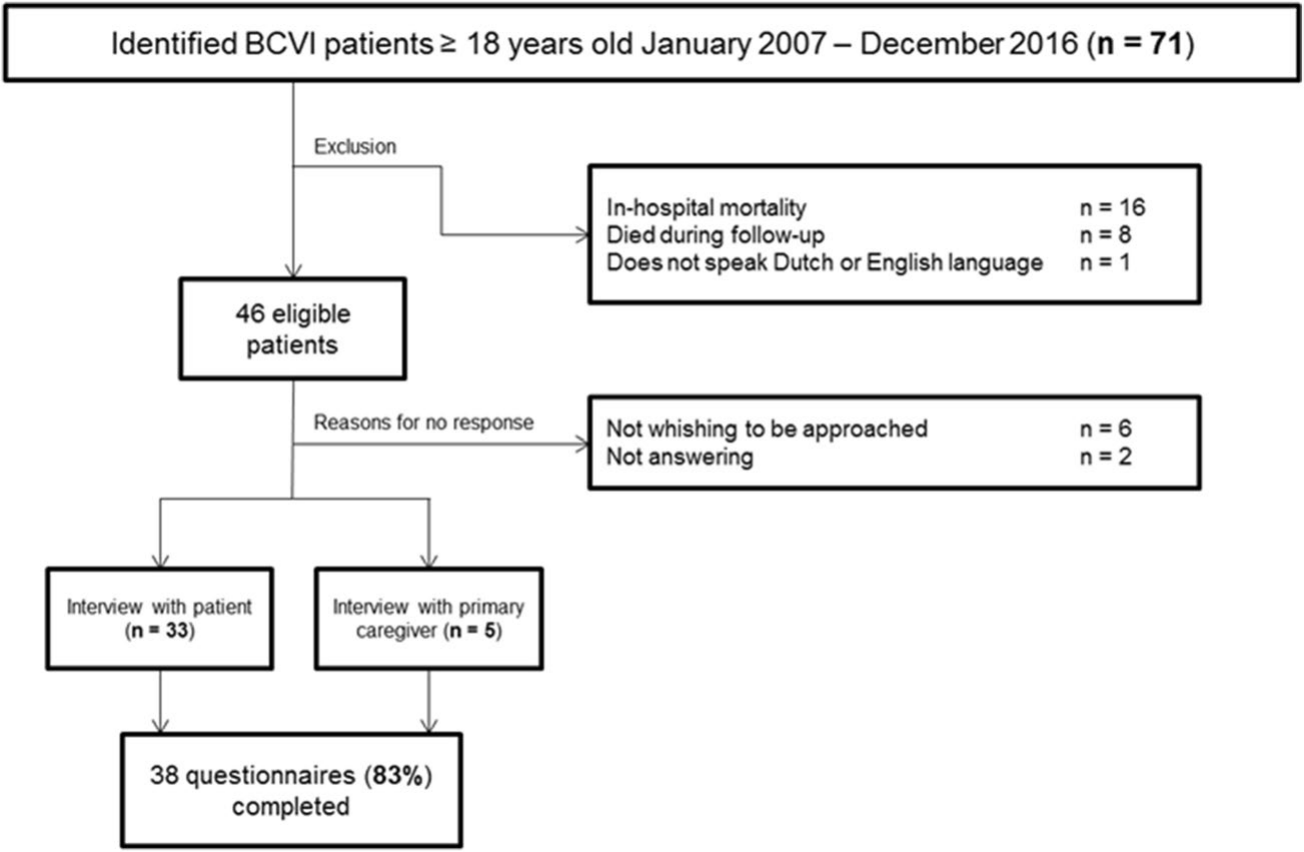
Flowchart displaying exclusion and response for EQ-6D™ questionnaire by telephonic interview

Out of the 71 identified BCVI patients, 46 (65%) patients were eligible for follow-up (Fig. 4). Thirty-eight completed the EQ-6D™ questionnaire (response rate of 83%). Median (IQR) time in years from trauma until follow-up was 4.0 (1.9–6.5). Mean (SD) age of survivors at follow-up in 2018 was 53 years (18). Five interviews were conducted with the primary caregiver, due to great difficulty with speaking (2 patients) or admission to a nursing home (3 patients).

### Functional outcomes

**Fig. 5:**
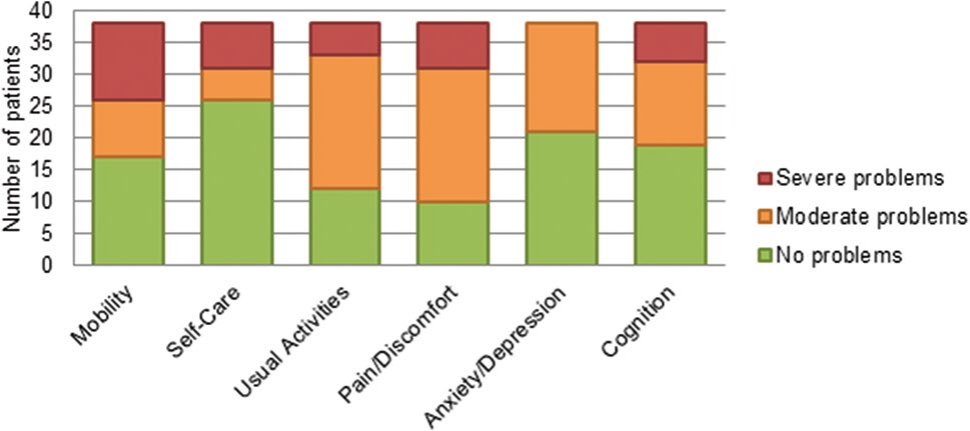
EQ-6D™ questionnaire reported problems of the studied BCVI population (*n* = 38)

Thirty-two responders (84%) reported having moderate or severe problems on any of the six dimensions of the EQ-6D™ questionnaire. The responses to all other dimensions in the EQ-6D™ questionnaire are shown in Fig. 5. The median EQ-5D™ index score and EQ-VAS™ were 0.69 (IQR 0.20–0.84) and 75 (IQR 50–85), respectively. The EQ-5D™ index score of BCVI patients was significantly lower (*p* < 0.001) compared the Dutch EQ-5D™ population index norm, as well as general trauma population presenting at our institution (*p* < 0.01), as shown in Table 4 [18, 19]. To assess the effect of ischemic stroke and associated brain injury on the overall functional performance of patients, functional outcomes on all of the EQ-6D™ domains of patient with and without ischemic stroke as well as patients with and without associated brain injury were compared. Patients suffering from ischemic stroke reported significantly more problems with mobility (*p* = 0.04) and self-care (*p* = 0.03). Patients suffering from associated brain injury reported significantly more cognitive problems (*p* = 0.03). These results are depicted in Tables 5 and 6.

**Table 4:** Mean (SE) {SD} EQ-5D™ index scores in comparison with reference populations

	Reference	BCVI patients	*P* value
Dutch population	0.89 (0.014)^a^	0.56 (0.057)	< 0.001*
General trauma population UMCU^b^	0.68 {0.26}	0.56 {0.35}	0.005*

^a^EQ-5D population index norm for people between age 45 and 54 (SE).^18^ Median (IQR) and mean {SD} age of the studied BCVI population was found to be 54 (37–69) and 53 {18}

^b^EQ-5D index score for general trauma population presenting at the same institution (Utrecht University Medical Center), derived from Gunning et al. [18]

*Significant differences are marked

**Table 5:** Comparison of functional outcomes by the presence or absence of ischemic stroke

Experienced problems (EQ-6D™ domain)	No Ischemic Stroke (*n* = 30)	Ischemic stroke (*n* = 8)	*P* value
Mobility	14 (47)	7 (88)	0.039*
Self-care	7 (23)	5 (63)	0.034*
Usual activities	19 (63)	7 (88)	0.191
Pain/discomfort	22 (73)	6 (75)	0.924
Anxiety/depression	14 (47)	3 (38)	0.643
Cognition	14 (47)	5 (63)	0.426

Data are presented as number of patients experiencing problems in a specific EQ-6D domain, and as percentage (%) of patients without and with ischemic stroke

*Significant differences are marked

**Table 6:** Comparison of functional outcomes by the presence or absence of brain injury

Experienced problems (EQ-6D™ domains)	No brain injury (*n* = 28)	Brain injury (*n* = 10)	*P* value
Mobility	15 (54)	6 (60)	0.726
Self-care	8 (29)	4 (40)	0.505
Usual activities	18 (64)	8 (80)	0.359
Pain/discomfort	22 (79)	6 (60)	0.252
Anxiety/depression	13 (46)	4 (40)	0.726
Cognition	11 (39)	8 (80)	0.027*

Data are presented as number of patients experiencing problems in a specific EQ-6D domain, and as percentage (%) of patients without and with brain injury

*Significant differences are marked

## Discussion

In this retrospective cohort study, we demonstrated the increasing incidence of diagnosed BCVIs among blunt trauma patients in a European level 1 trauma center. Improvements in CTA-scanning appear to result in more BCVIs diagnosed annually in the 10-year study period at our institution. Mortality is high and ischemic stroke is more frequently seen in patients without antiplatelet therapy. BCVI patients experience significantly more functional impairment at long-term follow-up in comparison to the general Dutch population, but also in comparison to the general trauma population. The presence of ischemic stroke was identified as a major cause for functional impairment.

### Incidence

Digital Subtraction Angiography (DSA) of the four cervical arteries remains the golden standard for diagnosing BCVI [6, 8, 20–23]. Unfortunately this is a time consuming, invasive and potentially harmful procedure. Since most BCVI patients have associated injuries in different regions of the body, which justify (total body) CT-scanning, CTA-scanning has become the diagnostic modality of first choice for screening patients at the emergency department. Studies examining the diagnostic value of CTA-scanning for diagnosing BCVI have had mixed results [24]. However, CTA-scanning is regarded as the best accessible and relatively accurate method for patients with a high probability of BCVI due to associated injuries.

In our institution, before 2010, trauma patients generally received CTA-scanning using a 64-slices scanner. BCVI incidence in period I was found to be 0.3% in the whole blunt trauma population, 0.9% in the polytrauma subgroup, 1.2% in patients with a basilar skull fracture and 4.6% in the cervical spine trauma subgroup. In period III, trauma patients received 256-slices CTA-scanning. In addition, to improve the diagnostic yield for cervical vascular injuries, the intravenous contrast administration flow for total body CT-scanning was increased from 3 to 6 ml/s and patients not eligible for total body CT-scanning received additional CTA-scanning in case of traumatic cervical CT-scan abnormalities. This resulted in an increase of found incidence in the whole blunt trauma group to 0.8%, the polytrauma subgroup 2.4%, patients with basilar skull fracture 1.9% and patients with cervical spine trauma 8.5%. Furthermore, it is a possibility that increased physician awareness has also been a contributing factor to diagnose BCVIs in the 10-year period. Nonetheless, we still believe that the reported incidence of BCVI in this study is an underestimation of the actual incidence due to the retrospective design of the study.

In addition, the predominant injury grading score according to Biffl et al. found at our institution was grade II (41%), followed by grade IV (35%). Intimal irregularities, described as Biffl et al. grade I, are reported to be the most frequent BCVIs [6, 7]. It should be noted that the Biffl et al. grading scale was based on angiography results and is, therefore, not validated for CTA-scanning [7, 25]. These injuries are generally self-healing and carry a relative low risk of ischemic stroke, thus considered less clinically relevant. Grade II injuries on the other hand, carry a larger potential to cause ischemic stroke and are, therefore, considered clinically important. We found that grade I BCVI only made up 11% of the total number of BCVIs when only CTA-scanning is performed.

### Functional outcomes

In many cases, BCVI was caused by high-energy cervical trauma and associated with severe (brain) injuries. The pivotal factor shaping the quality of life in BCVI patients at discharge is, apart from associated brain injury, the existence of neurological impairment due to ischemic cerebrovascular events. It is thought that asymptomatic BCVI patients have increased risk of ischemic cerebrovascular events compared to the general population. Whether this is caused by suboptimal antithrombotic therapy or a late effect of endovascular tissue damage remains unclear [7, 26–29]. Although small in numbers, treatment of BCVI using antiplatelet therapy in our population showed a significant effect on in-hospital stroke rate reduction (*p* < 0.01).

As our data suggest, a significant decline in function in daily life is also seen at long-term follow-up (median follow-up: 4 years after trauma), when compared to a Dutch reference population of the same age as well as to the general trauma population presenting at our institution.

As ischemic stroke and associated brain injury were likely to have great influence on functional outcomes of BCVI patients, we compared functional outcomes on each of the EQ-6D™ domains in patients with and without ischemic stroke and brain injury. Patients suffering from ischemic stroke reported significantly more problems with mobility and self-scare in comparison to patients without ischemic stroke. Patient suffering from brain injury reported significantly more cognitive problems in comparison to patients without brain injury. These results show the major role of ischemic stroke in long-term functional impairment and underscore the importance of prevention and treatment. Furthermore, a substantial amount of patients reported problems when performing usual activities and reported significant pain. As this can not only be contributed to ischemic stroke or traumatic brain injury, this may be caused by (severe) concomitant injuries.

### Strengths/limitations

Although many studies focus on screening for and treatment of BCVIs, limited data are available about long-term functional outcome. The longest study available on functional outcome following blunt cerebrovascular injury with a mean follow-up of 35 months reported similar adverse neurological events [2]. In addition, the findings of DiCocco et al. were significantly overshadowed by the low response rate of 37% [2]. The high percentage of patients included in our final analysis for follow-up (83%) facilitates an accurate comparison of functional outcome between BCVI patients and reference populations. Furthermore, the prospectively maintained institutional trauma database allowed us to accurately calculate the fluctuations in BCVI incidence over a 10-year period among multiple subgroups.

The limitations of this study make it necessary to use caution in extrapolating the data to the clinical field. First, the retrospective nature of the study may have resulted in missed BCVIs, which could lead to an underestimation of the BCVI incidence. Second, despite the relatively low drop-outs, patients in the non-responders group ought to be severely injured, which might have led to an overestimation of the functional outcomes compared. Furthermore, differences in treatment between the responders and the non-responder were noted, which may have affected the results. Third, we were unable to determine post-discharge ischemic strokes for patients who died during follow-up, as most of these patients did not undergo autopsy (not mandatory in our country). This, again, might have led to an overestimation of the functional outcomes.

## Conclusion

In conclusion, improved diagnostic modalities has increased BCVI incidence in trauma population subgroups during the 10-year study period. BCVI patients experience significantly more functional impairment as what could be expected in the Dutch population of the same age, as well as the general trauma population. Presence of ischemic stroke is a major cause of long-term functional impairment and more frequently seen in patients without antiplatelet therapy.

## Abbreviations

BCVI: Blunt cerebrovascular injury
CT: Computed tomography
CTA: Computed tomography angiography
EQ-6D™: EuroQol six-dimensional
EQ-5D™: EuroQol five-dimensional
PACS: Picture archiving and communicating system
ICD: International classification of diseases
ISS: Injury severity score
GCS: Glasgow coma scale
ICU: Intensive care unit
EQ-VAS™: EuroQol visual analog scale
IQR: Interquartile range
SD: Standard deviation
DSA: Digital subtraction angiography
SE: Standard error
UMCU: University medical center utrecht
OR: Odds ratio
